# Costs of Maternal Health-related Complications in Bangladesh

**DOI:** 10.3329/jhpn.v30i2.11315

**Published:** 2012-06

**Authors:** Mohammad Enamul Hoque, Timothy Powell-Jackson, Sushil Kanta Dasgupta, Mahbub Elahi Chowdhury, Marge Koblinsky

**Affiliations:** ^1^icddr,b, GPO Box 128, Dhaka 1000, Bangladesh; ^2^Health Economics and Financing Programme, London School of Hygiene & Tropical Medicine, London, UK; ^3^John Snow Inc., Arlington, Virginia, USA

**Keywords:** Healthcare costs, Health financing, Health shocks, Maternal health, Bangladesh

## Abstract

This paper assesses both out-of-pocket payments for healthcare and losses of productivity over six months postpartum among women who gave birth in Matlab, Bangladesh. The hypothesis of the study objective is that obstetric morbidity leads women to seek care at which time out-of-pocket expenditure is incurred. Second, a woman may also take time out from employment or from doing her household chores. This loss of resources places a financial burden on the household that may lead to reduced consumption of usual but less important goods and use of other services depending on the extent to which a household copes up by using savings, taking loans, and selling assets. Women were divided into three groups based on their morbidity patterns: (a) women with a severe obstetric complication (n=92); (b) women with a less-severe obstetric complication (n=127); and (c) women with a normal delivery (n=483). Data were collected from households of these women at two time-points—at six weeks and six months after delivery. The results showed that maternal morbidity led to a considerable loss of resources up to six weeks postpartum, with the greatest financial burden of cost of healthcare among the poorest households. However, families coped up with loss of resources by taking loans and selling assets, and by the end of six months postpartum, the households had paid back more than 40% of the loans.

## INTRODUCTION

When women give birth, they are at risk of maternal complications. Such complications can be both unpredictable and severe (1). In the absence of specialized care, the health of the mother and baby may rapidly deteriorate. The consequences of maternal complications, however, may not be limited to their immediate health impact. Women with obstetric complications may suffer consequences in terms of other problems, such as financial hardship, psychological distress, and longer-term health problems. In this series of papers investigating the consequences of maternal complications in Bangladesh, we focus on the repercussions in terms of economy of the household.

Economic consequences of maternal complications may be felt through a number of ways. First, poor health is likely to be associated with increased out-of-pocket (OOP) expenditure on medical care, thereby depleting household resources that might otherwise have been spent on consumption of goods. Second, poor health may lead to a loss of labour and, as a result, reduce household income. Any expenditure due to maternal complications has an impact on the total expenditure usually made by household members since resources are typically pooled from within a household. Thus, expenditure due to health shocks specific to one family member can affect the welfare of other family members.

A number of studies have explored household OOP expenditure on prenatal care (2) and on care received at childbirth, including care-seeking (3-6). Studies that focused on this question in South Asian countries tended to find OOP expenditure for maternal healthcare as high, both in absolute terms and compared to household income, which deters women from seeking healthcare (7-9). To-date, however, few studies explored longer-term costs associated with maternal ill-health and its impact on the economy of the household. Very little is known about whether costs continue to be incurred after childbirth and if these costs constitute a substantial economic burden on households. This information is important for understanding the full economic implications of poor maternal health and informing potential policy interventions to help families cope up with the situation.

In this paper, we addressed three issues. First, we determined the costs associated with maternal morbidity. Second, we sought to understand how households cope up with any loss of resources. The hypothesis behind these objectives is that obstetric complications lead women to seek care at which time OOP expenditure is incurred. Women may also take time out from employment or for doing their household chores when ill. The resulting loss of resources places a financial burden on the household, which may lead to the household's cutting back on consumption of usual but less important goods and use of other services depending on the extent to which they can resort to coping strategies.

Finally, this paper examined the economic consequences of maternal illness over six months postpartum in the context of rural Bangladesh. Costs were compared among three groups of women—those with normal births, those with severe complications, and those with less-severe complications. The findings have implications for the financing of maternal healthcare in Bangladesh where the Government has piloted an innovative demand-side voucher scheme targeting the poorest women at childbirth initiated after this study.

## MATERIALS AND METHODS

### Study site and study type

Data for the study were collected as part of a larger project which aimed at examining the burden of maternal ill-health and its programmatic implications. The study was conducted in Matlab, a rural area in Bangladesh and a study site of the International Centre for Diarrhoeal Disease Research, Bangladesh (icddr,b). In Matlab, the Community Health Research Workers (CHRWs) visit all married women in their homes every second month for data-collection while a second group of CHRWs distribute contraceptives and provide vaccines, oral rehydration solutions, vitamin A capsules, and nutrition education from their homes once a week. Four health subcentres run by icddr,b are located in the service area, each providing basic health services to a population of 20,000-30,000. Normal delivery care and basic essential obstetric care are provided in these health subcentres with back-up basic obstetric care provided by the Matlab Hospital of icddr,b. Women in need of comprehensive emergency obstetric care services are referred from the health centres and Matlab Hospital of icddr,b to either the government District Hospital or to private clinics in Chandpur district town. The proportion of women who gave birth at a health facility in the study area was 61.2% in 2007 (10), considerably higher than the 15% found in the country as a whole (11).

A prospective cohort study design was used for assessing the economic outcomes of women with a maternal complication compared to a group of women with normal delivery. A CHRW followed up each study woman who gave birth to document the place of childbirth. Two physicians then collected the medical records of the women who gave birth in a hospital, to examine whether they had a normal birth or were hospitalized for complications, either post-abortion or postpartum, over a two-year period (January 2007–December 2008). Every woman who had an obstetric complication (not related to abortion) in one of the local hospitals was enrolled in the study. A comparison group was selected through random sampling of women who gave birth without obstetric complications, irrespective of the place of delivery.

The women were then categorized into one of three groups based on their morbidity patterns: (a) women with a severe obstetric complication; (b) women with a less-severe obstetric complication; and (c) women with a normal delivery. Those with a severe maternal complication included women with major obstetric interventions due to absolute maternal indications, haemorrhage, hypertensive disorders of pregnancy, septic shock or septicaemia, and severe anaemia. The less-severe complication group included women who did not meet the criteria for severe maternal complications but hospital-records reported a diagnosis of vaginal bleeding, dystocia, hypertension, infection, and mild or moderate anaemia. All other women who did not meet the criteria for severe or less-severe maternal complications during hospitalization and gave birth vaginally or at home were included in the normal delivery group. Women who had an elective caesarean section were excluded from the analysis. A description of the three groups is detailed in Table 1.

### Sample-size and data-collection

Data were collected from households at two points in time—at six weeks and six months after delivery. Women selected for the study were contacted after extracting their current address from the surveillance registry with the CHRWs. The primary respondent of the interview was the head of the household as s/he manages the finances of the household and is, thus, responsible for most financial decisions. In the absence of the household head, the primary respondent was the person most informed about the finances of the family. Four trained female interviewers interviewed the respondents, using pretested structured questionnaire. Informed written consent was taken from the respondent before conducting the interview.

**Table 1.** Descriptive statistics on women with maternal complications and those with normal delivery

Descriptive statistics on women	Severe complication	Less-severe complication	Normal delivery
Delivered in a health facility (1=yes)	0.96	1.00	0.52
	(0.91-1.00)	-	(0.48-0.57)
Delivered in a private hospital (1=yes)	0.62	0.75	0.02
	(0.52-0.72)	(0.67-0.82)	(0.01-0.03)
Annual household consumption (Tk)	169,324	163,653	128,065
	(133981-204666)	(134471-192836)	(112459-143672)
Monthly household income (Tk)	18,730	17,813	12,287
	(13111-24349)	(14928-20698)	(10679-13895)
Age (years)	25.79	25.63	25.76
	(24.57-27.02)	(24.67-26.59)	(25.24-26.27)
Muslim (1=yes)	0.87	0.82	0.89
	(0.80-0.94)	(0.75-0.89)	(0.87-0.92)
Household members (1=yes)	6.23	6.26	5.97
	(5.76-6.70)	(5.84-6.68)	(5.76-6.17)
Education: 1-3 year(s) (1=yes)	0.04	0.02	0.05
	(0.00-0.09)	(-0.01-0.04)	(0.03-0.07)
Education: 4-7 years (1=yes)	0.34	0.17	0.38
	(0.24-0.43)	(0.11-0.24)	(0.33-0.42)
Education: 8 years or more (1=yes)	0.51	0.66	0.37
	(0.41-0.61)	(0.58-0.74)	(0.33-0.41)
Dry season (1=yes)	0.22	0.34	0.26
	(0.13-0.30)	(0.26-0.42)	(0.22-0.30)
Wet season (1=yes)	0.27	0.28	0.24
	(0.18-0.36)	(0.20-0.35)	(0.21-0.28)
Total nos.	92	127	487

Figures in parentheses indicate 95% confidence intervals. Descriptive statistics are not shown for ‘no education’ and ‘normal season’

Data were collected on household characteristics, family earnings, healthcare expenditure during childbirth and the postpartum period (up to 6 months), and the sources of finance to pay for maternal care. Data were also collected on the monthly income of the household and the women. The household monthly income was used for determining the household quintile. Information was also collected on the monthly expenditure of the household. In total, 786 households were successfully interviewed at six weeks (115 deliveries with severe complications; 139 deliveries with less-severe complications; and 532 with normal deliveries). Some women from the initially-selected samples were lost to follow-up during the second wave of data-collection at six months postpartum; 92, 127, and 483 in the three respective groups were available and included in the final analysis.

The analysis of health spending was disaggregated into two components: OOP expenditure and indirect OOP expenditure. Information on OOP expenditure relating to maternal healthcare was collected by asking the household head of the family about both direct and indirect OOP expenditure. Healthcare expenditure was measured comprehensively, including medical spending in the health facility (consultation fee, drugs, medical supplies, laboratory tests, inpatient costs, and theatre costs), medical spending incurred outside the facility (drugs and medical supplies), and related non-medical spending (transport, food, and hotel costs of relatives). For women who delivered at home, we captured expenditure in categories that remained relevant. Indirect OOP expenditure refers to spending relating to attendants and relatives of patients, mainly the family members of the patient, such as husband, mother, mother-in-law, or sister. The total cost was then calculated by adding the two components.

An analysis of the loss of productivity of the women, calculated as the product of the number of days lost due to illness and the wage rate of the patient, was done. The women were asked about the number of days they were absent from their work—paid or unpaid. The number of the total days lost was calculated by combining the days lost during delivery, days lost due to complication after delivery, and days lost due to neonatal complications. If a woman was absent, she was asked about who took care of her day-to-day activities during her absence, and if the person who took care of the day-to-day activities were paid, and if yes, then how much. Finally, the wage of the employed women was considered their wage rate. For housewives, a minimum market wage rate was considered their wage rate. Once the wage rate of the mother was decided, the wage rate was multiplied by the number of days absent from her day-to-day activities. The total, thus, gave productivity loss of these women for being absent from their work due to maternal complication.

The unit of analysis was the household. All financial data are presented in Taka (US$ 1=Tk 69 as in 2009). Data were entered into the Oracle 10g relational database software and Developer 6i and were analyzed using the Stata software (version 8).

## RESULTS

Table 1 presents the descriptive statistics relating to the study sample. The healthcare-seeking pattern at childbirth varied quite considerably between the groups. The large majority of women with an obstetric complication delivered in a private hospital. In contrast, very few women who gave birth without any complication in a health facility did so in a private hospital. The private sector catered almost exclusively for women in need of emergency care. The results in the remaining part of the table suggest that there are differences among the three groups in their background characteristics. Households in which the woman had a complication had a higher annual household expenditure and monthly income than the normal delivery group. Moreover, there were marked differences in the level of a woman's education. Just over half of those with a severe complication and almost two-thirds of the women in the less-severe group had attained grade 8 or higher while only just over one-third of the women with a normal delivery had the same level of education.

Table 2 shows the OOP expenditure on maternal healthcare incurred from the time of childbirth through six weeks postpartum and between six weeks and six months postpartum. The estimates showed that women with maternal complications (both severe and less severe) had much higher OOP expenditure compared to women with a normal delivery—over Tk 18,000 (US$ 261) vs about Tk 2,000 (US$ 29). The cost for women with less-severe complications was greater than for those with severe complications by about Tk 2,000. The reason may be due to the small sample-size of these cases (92 severe complicated cases and 127 less-severe complicated cases); this difference was not significant. Note also that even a normal delivery was not without cost.

This table also shows the trend in total household expenditure at different time periods—six weeks and six months postpartum. The household spending recorded for the period from childbirth through six weeks postpartum was driven by delivery-related costs. The household's spending on maternal healthcare fell dramatically in the subsequent period. At six months postpartum, the household's spending by those who suffered severe complications remained higher than the less-severe complication and normal delivery groups of women.

**Table 2.** Average total costs (in Taka) per woman for maternal care by the morbidity group, 2008-2009 (US$1=Tk 69 as in 2009)

Morbidity group	6 weeks	6 months	Total
Severe complication	17,395	1,134	18,529
(95% CI)	(14977-19812)	(570-1698)	(15857-21061)
Less-severe complication	19,418	702	20,120
(95% CI)	(17568-21267)	(473-930)	(18139-22115)
Normal delivery	2,139	650	2,789
(95% CI)	(1937-2342)	(511-789)	(2504-3035)

The period of analysis covered the time from childbirth to six months postpartum; CI=Confidence interval

Table 3 shows the medical spending on maternal healthcare and loss of productivity limited to the period from childbirth through six months postpartum. The loss of productivity of women was higher in women of the complicated group than that of the normal delivery group. It can also be seen from the table that the loss of household resources per woman due to delivery care was the highest among the women in the severe (total Tk 20,815) and less-severe groups (Tk 22,194) compared to the normal delivery group (Tk 3,763).

**Fig. 1.**
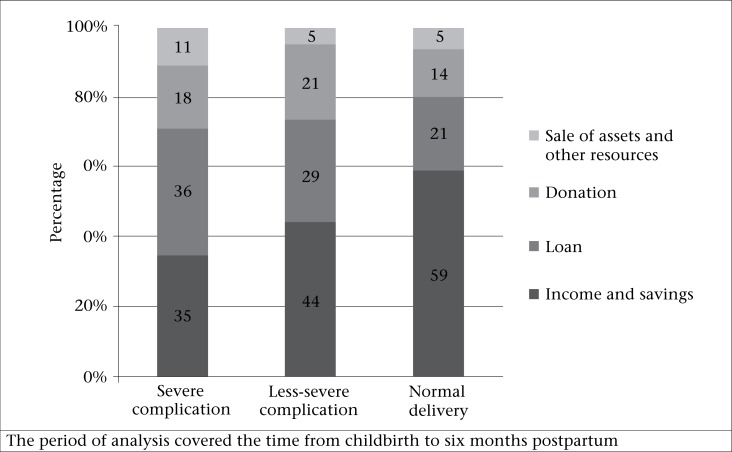
Sources of financing out-of-pocket payments on healthcare of women by morbidity group, 2008-2009

Figure 1 shows how the households financed their OOP payments for maternal healthcare. The sources of financing included income and savings, loans, donations, sale of assets, and other miscellaneous channels. The results showed that 35% of OOP payments for healthcare of the women in the severely-complicated group was financed mostly through income and savings; 36% was financed through loans without interest and 18% through donations. In the less-severe group, the financing was managed 44% through income and savings, 29% from loans, and 21% from donations. The normal delivery group paid around 59% of healthcare cost from their income and savings, followed by 21% from loans. The households in which the woman had a complication relied more on borrowing and donations than women with a normal delivery.

**Table 3.** Average loss of resources (in Taka) by morbidity group, including costs for maternal care plus productivity lost, 2008-2009 (US$1=Tk 69 as in 2009)

Morbidity group	Maternal care	Lost productivity of women	Total
Severe complication	18,529	2,286	20,815
(95% CI)	(15857-21061)	(1648-2923)	(17841-23648)
Less-severe complication	20,120	2,074	22,194
(95% CI)	(18139-22115)	(1700-2448)	(19712-24022)
Normal delivery	2,789	974	3,763
(95% CI)	(2504-3035)	(913-1034)	(3478-4064)

The period of analysis covered the time from childbirth to six months postpartum; CI=Confidence interval

**Fig. 2.**
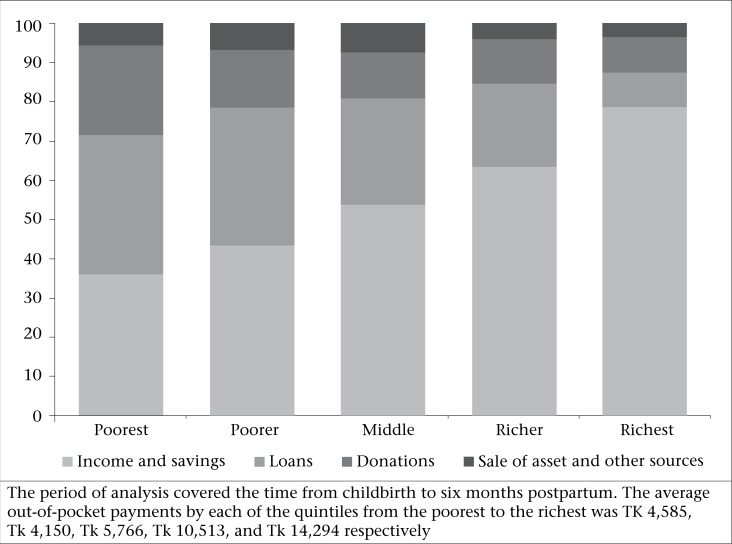
Proportion of out-of-pocket payments financed by each source according to the income quintile, 2008-2009

A clear pattern emerged when we analyzed the sources of finance to pay for OOP expenditure by the income quintile (Fig. 2). The richer households favoured income and savings as the main sources of funding. This may have a knock-on effect in terms of reduced household consumption but it suggests that such behaviour is largely discretionary. For example, the households in the richest quintile relied on income and savings to finance almost 80% of OOP expenditure while the households in the poorest quintile used these sources to fund less than 40% of such expenditure. As we moved from the poorest to the richest group, it was clear that dependence on loans and donations as the sources of income fell as a proportion of total spending. Interestingly, a small portion of the maternal healthcare expenditure came from the sale of assets, irrespective of whether the household was rich or poor.

Table 4 provides information on the loans paid back in six months. The household with severe complications took the maximum average amount of loan compared to the households with a woman with a less-severe complication or those with normal delivery. Again, the households with women with a normal delivery borrowed less for delivery- related purposes as they spent less on delivery and in the postpartum period. The households of women with less-severe complicated delivery paid back more of their loans than the households of women with severe complications.

Table 5 shows the total assets sold by each group. The value of assets sold by the severely-complicated group was the highest among the three groups. They sold both productive and non-productive assets (Table 5). Productive assets included land and livestock while non-productive assets comprised possessions of households, personal ornaments and jewelry. The households of women with less-severe complication sold more non-productive assets while those in the normal delivery group sold more productive assets.

## DISCUSSION

The study determined the long-term costs relating to maternal healthcare in a rural setting from the time of delivery to six months postpartum. The household's spending on maternal healthcare was concentrated primarily up to six weeks postpartum and more specifically at the time of delivery. Women with either severe or less-severe complications had a large loss of resources compared to women who delivered normally—spending approximately 10 times that for normal care. Loss of productivity of a woman, no matter which group she was in, did not represent a substantial proportion of the overall cost. This was because, in the rural Bangladesh setting, the economic value placed on the work of women tended to be low. This is not to suggest, however, that their daily work has little value in a broader sense of the meaning.

**Table 4.** Average loan (in Taka) by morbidity group for maternal healthcare costs and payment status at six months postpartum, 2008-2009

Morbidity group	Loan paid	Loan outstanding (at 6 months)	Total loan
Severe complication	4,670	9,087	13,757
(95% CI)	(2776-6563)	(6383-11792)	(11073-16442)
Less-severe complication	6,326	5,748	12,073
(95% CI)	(4125-8527)	(3441-8055)	(9507-14641)
Normal delivery	1,027	2,055	3,082
(95% CI)	(764-1290)	(1166-2944)	(2152-4012)

The period of analysis covered the time from childbirth to six months postpartum; CI=Confidence interval

**Table 5.** Average value (in Taka) of assets sold by type of assets and morbidity group, 2008-2009

Morbidity group	Productive assets	Non-productive assets	Total
Severe complication	1,042.71	963.54	2,006.25
(95% CI)	(-125-2211)	(294-1633)	(653-3360)
Less-severe complication	307.63	574.04	881.67
(95% CI)	(-67-682)	(175-973)	(218-1545)
Normal delivery	291.38	60.661	352.04
(95% CI)	(-210-792)	(4-117)	(-154-858)

The period of analysis covered the time from childbirth to six months postpartum; CI=Confidence interval

The OOP spending was substantially higher for those households with women with severe and less-severe complications at delivery compared to normal deliveries. Given the measure of maternal illness, this finding most likely reflects a causal relationship and not the influence of socioeconomic factors, although women who visited the facilities with complications were from the higher socioeconomic groups. This may be due to the fact that poor women delivered at home even if they had complications during delivery. In addition, after delivery, the poor women might not visit a hospital unless a very severe complication arose. The households of women with any complication used more loans and sold more productive assets than those in the normal-delivery group. This finding suggests that these women may suffer longer-term consequences and need to borrow money or sell assets after using their savings.

The households of women with complications during delivery relied to a greater degree than normal cases on loans without interest and donations from the family and friends. The two main sources of payment in this rural area were ‘income and savings’ and loans. Although a significant portion of medical care for the households of women with complications was borne by the households from their income and savings, these households still needed to take loans and asked for donations. This finding implies that most households in Matlab have some financial support or have a strong social network to protect them from financial shock. Despite having the lowest cost to cover, the women who had normal delivery used earnings from the sale of productive assets to cover their costs to a much larger extent than the other two groups (about 83% for women with normal delivery compared to 52% for women with severe and 35% for less-severe complications). The rural households initially sold their livestock, such as fowl and goats. While these households managed to cope up with loss of resources by taking out loans and selling assets, it may lead some households to fall below the poverty-line or further. Especially when a household sells their productive assets, they may face difficulty to earn enough to eat.

### Limitations

The study had several limitations. First, although the analysis was done using the six-week and six-month dataset, not all data were collected exactly at six weeks or six months. Some six-week data, for example, were collected at 10 weeks due to field operations starting later than scheduled. Second, there was the possibility of recall bias during collecting related information. This was a major problem generally in recalling specific amounts paid out. Third, the Matlab study area is unique compared to other parts of Bangladesh as the icddr,b health facilities do not charge for services. Hence, the costs reflected in this paper are assumed to be on the low-end of what happens in other parts of Bangladesh.

Although the icddr,b's subcentres and hospital are not charging, officially or unofficially, for services, households still have to spend a substantial amount for delivery. There are a number of possible reasons for this. First, the icddr,b's subcentres do not always cover the non-medical OOP expenditure, such as transport, food, and costs for companions. Second, complicated deliveries are referred to either the government District Hospital in Chandpur or to private clinics or hospitals, which are rapidly growing in numbers. Even with these limitations, the data provide insights into the maternal cost relating to various complications and normal delivery.

### Conclusions

The findings of the study provide no indication of whether the welfare of families with severe and less-severe complications suffers more compared to families with normal deliveries due to economic pressure during the six-month period of the study. Even so, it is anticipated that the long-term shocks resulting from such expenditure for poorer families would benefit by greater financial protection for maternal complications. Borrowing money from the informal sector may be a disaster for these families. A government-sponsored voucher scheme that now covers expenditure for delivery and maternal care for poor women will likely help in these cases. The voucher scheme has its own limitations but it may protect poor families from financial losses to some extent and may help these families protect themselves from the hands of local money-lenders. Otherwise, expenditure could lead to borrowing from these money-lenders, thus putting lands and goods at risk.

Although the universal coverage to pay for maternal illness is desirable, the more target-oriented strategy using vouchers for the poor to cover the expenditure of maternal illness is likely to be more sustainable. Finally, financial protection is needed for the poorest to encourage the use of facilities for delivery and prevent families from impoverishment.

## ACKNOWLEDGEMENTS

The study was made possible through support provided by the Office of Health, Infectious Diseases, and Nutrition, Global Health Bureau, United States Agency for International Development (USAID). icddr,b acknowledges with gratitude the commitment of USAID to its research efforts.
